# Association Between Visceral Adiposity Index and Metabolic Syndrome and Its Components: Evidence From the PERSIAN Guilan Cohort Study

**DOI:** 10.1002/edm2.70341

**Published:** 2026-09-22

**Authors:** Negin Letafatkar, Ehsan Amini‐Salehi, Seyyed Mohammad Hashemi, Soheil Hassanipour, Maryam Jafari, Saeed Ghaffari, Farahnaz Joukar, Fariborz Mansour‐Ghanaei

**Affiliations:** ^1^ Gastrointestinal and Liver Diseases Research Center Guilan University of Medical Sciences Rasht Iran; ^2^ Cardiovascular Research Center Hormozgan University of Medical Sciences Bandar Abbas Iran

**Keywords:** abdominal obesity, cardiometabolic risk, HDL cholesterol, metabolic syndrome, triglycerides, Visceral Adiposity Index

## Abstract

**Background:**

The Visceral Adiposity Index (VAI) is proposed as a surrogate marker of visceral adipose dysfunction. We evaluated its association with metabolic syndrome (MetS) and its components in a large Iranian population.

**Methods:**

This cross‐sectional study included 10,520 participants from the PERSIAN Guilan Cohort Study. VAI was calculated using sex‐specific equations. MetS was defined according to Adult Treatment Panel III criteria. Multivariable logistic regression assessed associations between VAI and MetS, while ROC analysis evaluated the discriminatory ability of VAI for identifying MetS.

**Results:**

MetS was present in 40.7% of participants (24.5% of men, 54.7% of women). VAI was significantly higher in individuals with MetS than in those without [3.31 (IQR 2.40–4.69) vs. 1.64 (1.14–2.32); *p* < 0.01]. In the fully adjusted model, each one‐unit increase in VAI was associated with higher odds of MetS in the overall population (OR = 2.41; 95% CI: 2.31–2.51), with a stronger association in women than in men (OR = 3.20 vs. 1.92; *p* for interaction < 0.001). VAI showed good discriminatory ability for identifying MetS in the total population (AUC = 0.84; 95% CI: 0.83–0.84), men (AUC = 0.83; 95% CI: 0.81–0.84), and women (AUC = 0.84; 95% CI: 0.83–0.85). Optimal VAI cut‐off values were 2.42 in the overall population, 2.08 in men and 2.69 among women. VAI was most strongly associated with triglycerides and HDL‐C, while significant associations were also observed with elevated fasting plasma glucose (FPG) and blood pressure (BP).

**Conclusion:**

VAI was strongly associated with the presence of MetS in this large Iranian population and demonstrated good discriminatory ability. Although part of this association may reflect shared components between VAI and the MetS definition, its significant associations with elevated FPG and BP suggest a broader relationship with cardiometabolic risk. Prospective studies and external validation are warranted before clinical application.

## Graphical abstract

In 10,520 adults from the PERSIAN Guilan Cohort, higher Visceral Adiposity Index was strongly associated with metabolic syndrome, particularly in women, and demonstrated good discriminatory ability (AUC 0.84). Sex‐specific VAI cut‐offs may help identify individuals at increased metabolic risk.

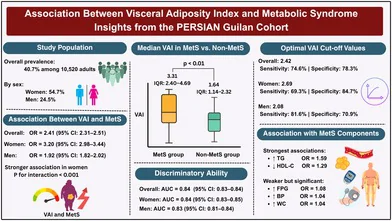

**Abbreviations**
ABSI: A Body Shape Index
ATP III: Adult Treatment Panel III
AUC: area under the curve
BMFI: Body Mass Fat Index
BMI: Body Mass Index
CMI: Cardiometabolic Index
DAI: Dysfunctional Adiposity Index
DBP: diastolic blood pressure
FPG: fasting plasma glucose
HDL‐C: high‐density lipoprotein cholesterol
HOMA‐IR: homeostatic model assessment of insulin resistance
IDF: International Diabetes Federation
JIS: joint interim statement
LAP: lipid accumulation product
MetS: metabolic syndrome
PCOS: polycystic ovary syndrome
ROC: receiver operating characteristic
SBP: systolic blood pressure
TG: triglycerides
TG/HDL‐C: triglyceride‐to‐HDL cholesterol ratio
VAI: Visceral Adiposity Index
WC: waist circumference
WHR: waist‐to‐hip ratio
WHtR: waist‐to‐height ratio

## 1 Introduction

Metabolic syndrome (MetS) is a multifactorial condition characterized by a cluster of metabolic abnormalities, including central obesity, insulin resistance, hypertension and dyslipidemia [1]. These interrelated factors significantly elevate the risk of developing cardiovascular diseases and type 2 diabetes [2, 3]. Globally, the prevalence of MetS among adults is estimated to range between 12.5% and 31.4%, with variations across different regions and populations [4]. This increasing prevalence poses a substantial burden on healthcare systems worldwide, contributing to heightened morbidity and mortality rates [5].

Central to the pathogenesis of MetS is visceral adipose tissue (VAT), which plays a critical role in metabolic dysfunction [6, 7]. Traditional anthropometric measures such as body mass index (BMI), waist circumference (WC) and waist‐to‐height ratio (WHtR) have been employed to estimate VAT [8]. However, these measures may not accurately reflect visceral fat distribution and function [9]. To address this limitation, indices that combine anthropometric and biochemical parameters, such as triglyceride (TG) and high‐density lipoprotein cholesterol (HDL‐C), have been developed, demonstrating improved ability to identify MetS compared to single‐parameter assessments [10].

The VAI, introduced by Amato et al. in 2010, is a novel, sex‐specific indicator that integrates BMI, WC, TG and HDL‐C to estimate visceral fat function and insulin sensitivity [11]. In their study, VAI was first modelled on 315 nonobese healthy subjects and retrospectively validated in 1498 primary care patients. The results demonstrated that VAI was independently associated with both cardiovascular and cerebrovascular events, and showed a significant inverse correlation with insulin sensitivity during euglycemic‐hyperinsulinemic clamp in a subgroup of patients. By contrast, no correlations were found for WC and BMI. These findings suggest that VAI is a valuable indicator of visceral adipose function and insulin sensitivity, and its increase is strongly associated with cardiometabolic risk [11].

Subsequent studies have corroborated the utility of VAI as a surrogate marker for visceral adiposity and its associated risks. For instance, research has demonstrated that VAI was developed as a surrogate marker of visceral adipose dysfunction, and its relationship with imaging‐based measures of visceral adiposity has subsequently been evaluated in different populations [11, 12]. Moreover, VAI has been associated with various health‐related outcomes, including hypertension, chronic kidney disease and type 2 diabetes, across different ethnic populations [13, 14, 15]. Despite the growing body of evidence supporting the clinical relevance of VAI, variations in its discriminatory ability across different populations and geographical regions have been observed.

In light of these findings, our study aims to assess the association between VAI and MetS within a large Iranian cohort comprising over 10,000 individuals. By analysing this substantial dataset, we seek to provide insights into the association between VAI and MetS in the Iranian population and its discriminatory ability within this study population. Our research endeavours to contribute valuable data to the existing literature, facilitating the development of targeted screening and intervention strategies to mitigate the burden of MetS.

## 2 Methodology

### 2.1 Study Design and Sampling

We conducted a cross‐sectional population‐based study utilizing baseline data from the PERSIAN Guilan Cohort Study (PGCS), which includes 10,520 individuals aged 35–70 years. The methodology and profile of this cohort have been previously described in detail [16, 17].

### 2.2 Data Collection and Measurements

Data for this study were obtained through a comprehensive, standardized protocol that included structured interviews, clinical examinations and laboratory testing, all carried out by trained healthcare staff. Information on demographics, socioeconomic status, and lifestyle factors was gathered using validated questionnaires. Variables collected included age, sex, marital status, level of education, employment status and urban or rural residency.

Physical activity was assessed using a validated questionnaire that recorded participants' daily activities over the past year. The metabolic equivalent (MET) values for each activity were obtained from a compendium of physical activities, and calculations were performed based on the reported activities over a 24‐h period. Additionally, average weekly MET minutes were collected, encompassing activities from leisure, work and sports [18]. Smoking and alcohol consumption were assessed through structured self‐reported questionnaires. The smoking status of participants was divided into smokers and non‐smokers based on their responses to a self‐reported question asking if they had smoked at least 100 cigarettes in their lifetime, with options of yes or no. The use of medications, including lipid‐lowering agents, was assessed through structured self‐report and verified by reviewing participants' baseline medication history.

Anthropometric measurements were performed following standard operating procedures [16]. Height was measured using a fixed stadiometer, and weight was measured with a calibrated mechanical scale. BMI was computed by dividing weight in kilograms by height in meters squared. WC was measured at the midpoint between the lowest rib and iliac crest using non‐elastic measuring tape. Blood pressure was measured using a mercury sphygmomanometer under resting conditions. Participants were seated in a quiet environment for at least 10 min before two readings were taken from each arm. The mean systolic and diastolic values were used in subsequent analyses [19].

After a minimum 12‐h overnight fast, venous blood samples were collected. Fasting plasma glucose (FPG), TG, total cholesterol (TC) and HDL‐C were measured using enzymatic colorimetric assays. These tests were conducted using automated analysers in a central certified laboratory. Low‐density lipoprotein cholesterol (LDL‐C) was calculated using the Friedewald formula for individuals with TG < 400 mg/dL.

### 2.3 Visceral Adiposity Index Calculation

VAI was calculated separately for men and women using sex‐specific equations that incorporate WC, BMI, TG and HDL‐C, with TG and HDL‐C expressed in mmol/L [11]:

- Men: VAI = (WC/(39.68 + 1.88 × BMI)) × (TG/1.03) × (1.31/HDL‐C)
- Women: VAI = (WC/(36.58 + 1.89 × BMI)) × (TG/0.81) × (1.52/HDL‐C)

For analyses in the total population, the resulting sex‐specific VAI values were analysed together as a single VAI variable; no additional standardization, transformation, or alternative non‐sex‐specific equation was applied. Sex was included as a covariate in adjusted models.

### 2.4 Definition of MetS and Its Components

MetS and its individual components were defined based on the ATP III criteria [20]: A diagnosis of MetS was made if at least three of the following were present:

1. Abdominal obesity (increased WC based on ATP III cut‐offs)
2. Elevated TG (≥ 150 mg/dL)
3. Reduced HDL‐C (< 40 mg/dL in men, < 50 mg/dL in women)
4. Elevated FPG (≥ 100 mg/dL or use of antidiabetic medications)
5. Elevated blood pressure (SBP ≥ 130 mmHg, DBP ≥ 85 mmHg, or use of antihypertensives)

### 2.5 Statistical Analysis

All statistical analyses were performed using R software (version 4.5.0). Descriptive statistics were used to summarize baseline characteristics of the study population. Continuous variables were summarized as mean ± SD or median with interquartile range according to their distribution. Categorical variables were presented as frequencies and percentages. Given the large sample size, distributional characteristics of continuous variables were assessed using graphical inspection and descriptive measures of skewness, and formal normality tests were interpreted cautiously. Between‐group comparisons were performed using independent‐samples *t*‐tests or Mann–Whitney *U* tests, as appropriate. Categorical variables were compared using the chi‐square test or Fisher's exact test, where appropriate.

There were no missing data for the main exposure and outcome variables. Participants with complete data for covariates included in each model were analysed.

To evaluate the association between the VAI and the presence of MetS, logistic regression models were constructed treating VAI as both a continuous variable and in quartiles (Q1–Q4), with Q1 as the reference category. For analyses in the overall population, VAI quartiles were defined according to the distribution of VAI in the total study population. For sex‐stratified analyses, quartiles were defined separately within men and women according to their respective sex‐specific VAI distributions. Odds ratios (ORs) with 95% confidence intervals (CIs) were calculated.

Four models of adjustment were applied:

- Crude model: No adjustment
- Model 1: Adjusted for age and sex
- Model 2: Further adjusted for lipid‐lowering drug use
- Model 3: Fully adjusted for age, sex, lipid‐lowering drugs, habitat (urban/rural), education level (years), alcohol use and cigarette smoking status

However, sex was omitted from models 1–3 in the sex‐stratified analysis.

To assess whether the association between VAI and MetS differed by sex, a formal interaction test between VAI and sex was added to the fully adjusted logistic regression model, and the corresponding *p*‐value was reported as the *p* for interaction.

To examine the association between VAI and each individual component of MetS, logistic regression analyses were performed following the same stepwise adjustment strategy applied for the overall MetS outcome, including the Crude model, Model 1, Model 2 and Model 3.

Receiver operating characteristic (ROC) curve analysis was conducted to evaluate the discriminatory ability of VAI for identifying MetS in the study population. The area under the ROC curve (AUROC) and its 95% CI were calculated. Optimal VAI cut‐off values were determined using Youden's Index, maximizing the sum of sensitivity and specificity. ROC curves were generated for VAI in the overall population and separately by sex. In addition, further ROC analyses were performed in the overall population to compare the discriminatory ability of VAI with other routinely available adiposity‐ and lipid‐related indices, including BMI, WC, WHtR and the TG/HDL‐C ratio.

Exploratory subgroup analyses were performed to examine the consistency of the association between continuous VAI and MetS across selected demographic and clinical strata, including sex, age, marital status, education, occupation and BMI categories. Subgroup‐specific crude ORs with 95% CIs were estimated using logistic regression and presented graphically in a forest plot. A two‐tailed *p*‐value of less than 0.05 was considered statistically significant in all analyses.

## 3 Results

### 3.1 Characteristics of the Study Population

The study included 10,520 adults from the PERSIAN Guilan cohort, of whom 4280 individuals (40.7%) met the diagnostic criteria for MetS as defined by ATP III criteria. The prevalence of MetS was 24.5% in men (*n* = 1198 out of 4887) and 54.7% in women (*n* = 3082 out of 5633), indicating a substantially higher burden among female participants (Table 1). VAI was significantly higher among participants with MetS compared with those without MetS. In the total population, median VAI was 3.31 (IQR: 2.40–4.69) in individuals with MetS and 1.64 (IQR: 1.14–2.32) in those without MetS (*p* < 0.01). This pattern was consistent in both men and women, as shown in Table 1.

**TABLE 1:** Demographic, clinical and lifestyle characteristics of participants stratified by MetS status.

Variables		Total (*n* = 10,520)			Men (*n* = 4887)			Women (*n* = 5633)		
		MetS + (*n* = 4280)	MetS – (*n* = 6240)	*p*	MetS + (*n* = 1198)	MetS − (*n* = 3689)	*p*	MetS + (*n* = 3082)	MetS − (*n* = 2551)	*p*
Age (mean ± SD, years)		53 ± 8.84	50.5 ± 8.80	< 0.01	52.9 ± 8.78	51 ± 8.84	< 0.01	53 ± 8.86	49.8 ± 8.7	< 0.01
Occupation	Yes	1724	4015	< 0.01	1017	3308	< 0.01	707	707	< 0.01
	No	2556	2225		181	381		2375	1844	
Marital status	Single	95	210	< 0.01	14	64	0.540	81	146	< 0.01
	Married	3790	5737		1165	3568		2625	2169	
	Widow/Widower	343	223		13	35		330	188	
	Divorced	52	70		6	22		46	48	
Residency type	Urban	1789	2824	< 0.01	397	1665	< 0.01	1392	1159	0.86
	Rural	2491	3416		801	2024		1690	1392	
Education	0–5 years of schooling	2356	2694	< 0.01	434	1407	0.193	1922	1287	< 0.01
	6–12 years of schooling	1727	3105		638	1952		1089	1153	
	University/College	197	441		126	330		71	111	
Physical activity (mean ± SD, MET)		39.1 ± 7.41	42.7 ± 9.5	< 0.01	40.8 ± 9.12	44.4 ± 10.2	< 0.01	38.5 ± 6.51	40.3 ± 7.65	< 0.01
Cigarette smoking	Yes	632	1952	< 0.01	591	1931	0.08	41	21	0.09
	No	3645	4284		604	1754		3041	2530	
Alcohol consumption	Yes	359	1156	< 0.01	355	1153	0.307	4	3	1.00
	No	3919	5084		843	2536		3076	2548	
BMI (mean ± SD)		30.3 ± 4.9	26.7 ± 4.66	< 0.01	29.0 ± 4.26	25.15 ± 3.74	< 0.01	30.8 ± 5.03	28.8 ± 5	< 0.01
WC (mean ± SD)		104.63 ± 11	94.82 ± 11.72	< 0.01	101.37 ± 10.5	91.1 ± 9.77	< 0.01	105.9 ± 10.95	100.24 ± 12.2	< 0.01
SBP (mean ± SD)		125 ± 17.3	114 ± 14.9	< 0.01	128 ± 16.8	116 ± 15.3	< 0.01	123 ± 17.3	111 ± 13.8	< 0.01
DBP (mean ± SD)		80.6 ± 10.8	74.5 ± 10.4	< 0.01	84.2 ± 10.6	75.9 ± 10.4	< 0.01	79.2 ± 10.6	72.5 ± 10	< 0.01
FPG (median, IQR)		102 [92–120]	91 [86–97]	< 0.01	106 [96–125]	92 [86–98]	< 0.01	91 [84–101]	85 [80–90]	< 0.01
TC (mean ± SD)		195 ± 42.4	191 ± 36.4	< 0.01	194 ± 41.4	190 ± 37	0.014	195 ± 42.7	193 ± 35.4	0.006
TG (median, IQR)		180 [137–237]	115 [87–148]	< 0.01	203 [163–266.3]	123 [90.5–171]	< 0.01	171 [127–225]	106 [82–133]	< 0.01
HDL (mean ± SD)		44.4 ± 9.75	51.1 ± 10.9	< 0.01	40.3 ± 9.08	48.5 ± 10.2	< 0.01	45.9 ± 9.55	54.9 ± 10.9	< 0.01
LDL (mean ± SD)		110.9 ± 33.97	114.18 ± 3 0.59	< 0.01	108 ± 34.4	113 ± 30.7	< 0.01	112 ± 33.8	115 ± 30.4	< 0.01
MetS components	High FPG	2340	1779	< 0.01	748	1152	< 0.01	1592	627	< 0.01
	Low HDL‐C	2379	1890	< 0.01	702	1099	< 0.01	1677	791	< 0.01
	High TG	2528	2009	< 0.01	835	1344	< 0.01	1693	665	< 0.01
	High WC	3020	3230	< 0.01	713	1553	< 0.01	2307	1677	< 0.01
	High BP	2194	1789	< 0.01	695	1161	< 0.01	1499	628	< 0.01
VAI (median, IQR)		3.31 [2.40–4.69]	1.64 [1.14–2.32]	< 0.01	3.09 [2.28–4.26]	1.51 [1.03–2.24]	< 0.01	3.41 [2.47–4.85]	1.80 [1.32–2.39]	< 0.01

Abbreviations: BMI, Body Mass Index; DBP, diastolic blood pressure; FPG, fasting plasma glucose; HDL‐C, high‐density lipoprotein cholesterol; LDL, low‐density lipoprotein; MetS, metabolic syndrome; SBP, systolic blood pressure; TC, total cholesterol; TG, triglycerides; VAI, Visceral Adiposity Index; WC, waist circumference; WSI, Wealth Status Index.

### 3.2 Association Between VAI and MetS: Logistic Regression Analysis

The association between the VAI and MetS was examined using logistic regression models, treating VAI as both a continuous and categorical variable. In the overall population, VAI as a continuous variable showed a strong positive association with the presence of MetS. In the crude model, each one‐unit increase in VAI was associated with an odds ratio (OR) of 2.42 (95% confidence interval [CI]: 2.33–2.52; *p* < 0.01). This association remained stable after sequential adjustment for potential confounders. After adjusting for age and sex (Model 1), the OR was 2.41 (95% CI: 2.31–2.52), and further adjustment for lipid‐lowering medication use (Model 2) resulted in an OR of 2.40 (95% CI: 2.30–2.51). In the fully adjusted model (Model 3), the association remained statistically significant (OR = 2.41; 95% CI: 2.31–2.51; *p* < 0.01), as shown in Table 2.

**TABLE 2:** Association between VAI and MetS in overall population, men and women.

Variable		Crude		Model 1		Model 2		Model 3	
		OR, 95% CI	*p*	OR, 95% CI	*p*	OR, 95% CI	*p*	OR, 95% CI	*p*
Overall	VAI (continuous)	2.42 (2.33–2.52)	< 0.01	2.41 (2.31–2.52)	< 0.01	2.40 (2.30–2.51)	< 0.01	2.41 (2.31–2.51)	< 0.01
	Quartile 1 (VAI: 0.242–1.40)	Ref	Ref	Ref	Ref	Ref	Ref	Ref	Ref
	Quartile 2 (VAI: 1.40–2.18)	2.88 (2.45–3.39)	< 0.01	2.49 (2.11–2.94)	< 0.01	2.44 (2.06–2.89)	< 0.01	2.45 (2.06–2.90)	< 0.01
	Quartile 3 (VAI: 2.18–3.35)	10.38 (8.90–12.09)	< 0.01	9.28 (7.93–10.9)	< 0.01	9.29 (7.92–10.9)	< 0.01	9.42 (8.01–11.08)	< 0.01
	Quartile 4 (VAI: 3.35–35.4)	40.98 (34.78–48.3)	< 0.01	40.9 (34.5–48.7)	< 0.01	40.7 (34.2–48.5)	< 0.01	41.35 (34.70–49.26)	< 0.01
Men	VAI (continuous)	1.87 (1.78–1.97)	< 0.01	1.94 (1.84–2.04)	< 0.01	1.93 (1.83–2.03)	< 0.01	1.92 (1.82–2.02)	< 0.01
	Quartile 1 (VAI: 0.242–1.18)	Ref	Ref	Ref	Ref	Ref	Ref	Ref	Ref
	Quartile 2 (VAI: 1.18–1.81)	3.68 (2.46–5.49)	< 0.01	3.69 (2.46–5.52)	< 0.01	3.57 (2.38–5.35)	< 0.01	3.69 (2.45–5.56)	< 0.01
	Quartile 3 (VAI: 1.81–2.85)	15.95 (11.00–23.13)	< 0.01	16.70 (11.49–24.27)	< 0.01	16.28 (11.19–23.69)	< 0.01	16.93 (11.57–24.78)	< 0.01
	Quartile 4 (VAI: 2.85–30.1)	48.03 (33.21–69.46)	< 0.01	54.99 (37.87–79.85)	< 0.01	53.74 (36.97–78.11)	< 0.01	55.16 (37.70–80.72)	< 0.01
Women	VAI (continuous)	3.09 (2.89–3.30)	< 0.01	3.17 (2.96–3.40)	< 0.01	3.17 (2.96–3.40)	< 0.01	3.20 (2.98–3.44)	< 0.01
	Quartile 1 (VAI: 0.289–1.67)	Ref	Ref	Ref	Ref	Ref	Ref	Ref	Ref
	Quartile 2 (VAI: 1.67–2.50)	2.16 (1.82–2.56)	< 0.01	2.14 (1.80–2.55)	< 0.01	2.14 (1.79–2.55)	< 0.01	2.16 (1.81–2.58)	< 0.01
	Quartile 3 (VAI: 2.50–3.76)	8.73 (7.35–10.36)	< 0.01	9.17 (7.68–10.94)	< 0.01	9.14 (7.63–10.95)	< 0.01	9.40 (7.84–11.27)	< 0.01
	Quartile 4 (VAI: 3.76–35.4)	53.51 (41.88–68.38)	< 0.01	58.26 (45.39–74.79)	< 0.01	58.73 (45.63–75.58)	< 0.01	60.53 (46.96–78.03)	< 0.01

Abbreviations: CI, confidence interval; OR, odds ratio; Ref, reference category; VAI, Visceral Adiposity Index.

When VAI was categorized into quartiles, compared to participants in the lowest quartile (Q1), those in Q2 had an adjusted odds ratio of 2.45 (95% CI: 2.06–2.90), while those in Q3 and Q4 had substantially higher odds of MetS, with ORs of 9.42 (95% CI: 8.01–11.08) and 41.35 (95% CI: 34.70–49.26), respectively (*p* < 0.01 for all). A graded increase in the odds of MetS was observed across VAI quartiles. These findings are detailed in Table 2.

Sex‐stratified analyses showed that the association between VAI and MetS remained significant in both men and women, with some differences in effect size. In fully adjusted models, each unit increase in VAI was associated with an OR of 1.92 (95% CI: 1.82–2.02) in men and 3.20 (95% CI: 2.98–3.44) in women. A formal interaction test showed a significant interaction between VAI and sex in relation to MetS (*p* for interaction < 0.001), supporting stronger association between VAI and MetS among women than in men. Similarly, the odds of MetS across VAI quartiles increased progressively in both sexes. Among men, the ORs for Q2, Q3 and Q4 compared to Q1 were 3.69, 16.93 and 55.16, respectively. Among women, these values were 2.16, 9.40 and 60.53, respectively (*p* < 0.01 for all comparisons). These sex‐specific results are also presented in Table 2.

### 3.3 Discriminatory Ability and Optimal Thresholds of VAI for Identifying MetS

**FIGURE 1:**
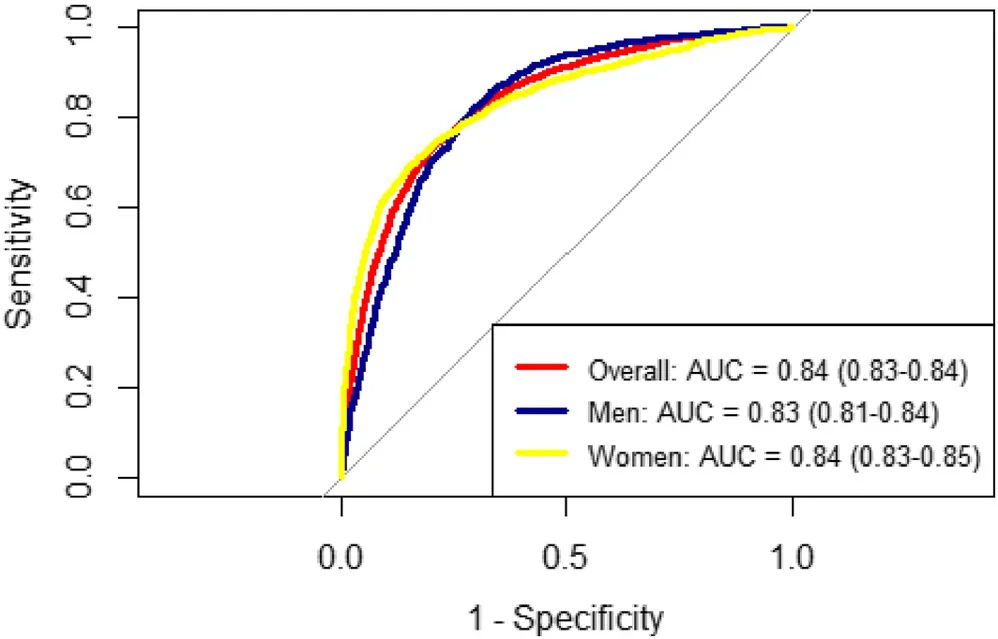
ROC curves for VAI in identifying MetS in the overall population, men and women.

The discriminatory ability of VAI for identifying MetS was assessed using ROC curve analysis in the overall population and separately by sex. In the total population, VAI showed good discriminatory ability, with an AUC of 0.835 (95% CI: 0.827–0.842). Similar discriminatory performance was observed in sex‐specific analyses, with an AUC of 0.826 (95% CI: 0.814–0.839) in men and 0.836 (95% CI: 0.825–0.846) among women (Figure 1, Table 3).

**TABLE 3:** Discriminatory ability of VAI and multivariable models for identifying MetS in the overall population, men and women.

Variable		AUC (95% CI)	Optimal cut off	Sensitivity	Specificity
Overall	Crude	0.835 (0.827–0.842)	2.42	0.746	0.783
	Model 1	0.853 (0.846–0.861)	—	0.746	0.798
	Model 2	0.861 (0.854–0.868)	—	0.755	0.809
	Model 3	0.863 (0.856–0.870)	—	0.766	0.801
Men	Crude	0.826 (0.814–0.839)	2.08	0.816	0.709
	Model 1	0.824 (0.811–0.837)	—	0.768	0.742
	Model 2	0.829 (0.817–0.841)	—	0.768	0.742
	Model 3	0.862 (0.829–0.884)	—	0.732	0.779
Women	Crude	0.836 (0.825–0.846)	2.69	0.693	0.847
	Model 1	0.85 (0.84–0.86)	—	0.768	0.792
	Model 2	0.859 (0.85–0.869)	—	0.744	0.825
	Model 3	0.862 (0.853–0.872)	—	0.784	0.791

*Note:* Crude AUC represents the discriminatory ability of VAI alone. Adjusted AUCs represent model‐based discrimination after inclusion of covariates. Cut‐off values were derived from the crude VAI‐only ROC analysis.

In model‐based ROC analyses, discrimination improved slightly after adjustment for covariates. In the overall population, the AUC was 0.853 (95% CI: 0.846–0.861) in Model 1, 0.861 (95% CI: 0.854–0.868) in Model 2 and 0.863 (95% CI: 0.856–0.870) in Model 3. In the fully adjusted model, the AUC was 0.862 (95% CI: 0.829–0.884) in men and 0.862 (95% CI: 0.853–0.872) in women (Table 3).

Optimal VAI thresholds were determined using Youden's Index. The optimal cut‐off value was 2.42 in the total population, with a sensitivity of 0.746 and specificity of 0.783. The corresponding cut‐off values were 2.08 in men, with a sensitivity of 0.816 and specificity of 0.709, and 2.69 in women, with a sensitivity of 0.693 and specificity of 0.847 (Table 3).

Additional ROC analyses were performed in the overall population to compare the discriminatory ability of VAI with other routinely available adiposity‐ and lipid‐related indices. VAI showed the highest discriminatory ability among the evaluated indices, with an AUC of 0.835 (95% CI: 0.827–0.842), followed by the TG/HDL‐C ratio (AUC = 0.775; 95% CI: 0.766–0.784), WHtR (AUC = 0.756; 95% CI: 0.747–0.765), WC (AUC = 0.737; 95% CI: 0.728–0.747) and BMI (AUC = 0.715; 95% CI: 0.706–0.725).

### 3.4 Association Between VAI and Individual Components of MetS

VAI was significantly associated with each individual component of MetS in the fully adjusted model. The strongest associations were observed for elevated TG and low HDL‐C, with each one‐unit increase in VAI associated with higher odds of elevated TG (OR = 1.59; 95% CI: 1.54–1.64; *p* < 0.01) and low HDL‐C (OR = 1.29; 95% CI: 1.26–1.32; *p* < 0.01). Smaller but statistically significant associations were also observed for elevated FPG (OR = 1.08; 95% CI: 1.06–1.10; *p* < 0.01), elevated WC (OR = 1.04; 95% CI: 1.00–1.06; *p* < 0.01) and elevated BP (OR = 1.04; 95% CI: 1.03–1.06; *p* < 0.01).

In quartile‐based analyses, the odds of all MetS components increased across higher VAI quartiles. Compared with participants in the lowest VAI quartile, those in the highest quartile had higher odds of elevated TG (OR = 13.12; 95% CI: 11.45–15.04), low HDL‐C (OR = 5.14; 95% CI: 4.54–5.82), elevated FPG (OR = 1.50; 95% CI: 1.33–1.68), elevated WC (OR = 1.39; 95% CI: 1.24–1.57) and elevated BP (OR = 1.40; 95% CI: 1.25–1.58), all with *p* < 0.01, as shown in Table 4. Because of the right‐skewed distribution of VAI and the broad range of values in the highest quartile, the estimates for Q4 should be interpreted cautiously.

**TABLE 4:** Association between VAI and components of MetS in whole population.

Variable		Crude		Model 1		Model 2		Model 3	
		OR (95% CI)	*p*	OR (95% CI)	*p*	OR (95% CI)	*p*	OR (95% CI)	*p*
Elevated FPG	VAI (continuous)	1.08 (1.061–1.10)	< 0.01	1.085 (1.065–1.105)	< 0.01	1.075 (1.055–1.095)	< 0.01	1.075 (1.056–1.095)	< 0.01
	Quartile 1 (VAI: 0.242–1.40)	Ref	Ref	Ref	Ref	Ref	Ref	Ref	Ref
	Quartile 2 (VAI: 1.40–2.18)	1.138 (1.016–1.274)	0.025	1.143 (1.019–1.282)	0.022	1.119 (0.997–1.256)	0.056	1.124 (1.001–1.261)	0.048
	Quartile 3 (VAI: 2.18–3.35)	1.367 (1.223–1.529)	< 0.01	1.392 (1.241–1.561)	< 0.01	1.346 (1.199–1.512)	< 0.01	1.355 (1.207–1.522)	< 0.01
	Quartile 4 (VAI: 3.35–35.4)	1.536 (1.374–1.718)	< 0.01	1.579 (1.407–1.773)	< 0.01	1.485 (1.322–1.668)	< 0.01	1.498 (1.332–1.684)	< 0.01
Low HDL‐C	VAI (continuous)	1.306 (1.276–1.337)	< 0.01	1.298 (1.268–1.33)	< 0.01	1.294 (1.264–1.325)	< 0.01	1.288 (1.258–1.319)	< 0.01
	Quartile 1 (VAI: 0.242–1.40)	Ref	Ref	Ref	Ref	Ref	Ref	Ref	Ref
	Quartile 2 (VAI: 1.40–2.18)	1.578 (1.397–1.784)	< 0.01	1.576 (1.394–1.782)	< 0.01	1.568 (1.386–1.773)	< 0.01	1.552 (1.371–1.756)	< 0.01
	Quartile 3 (VAI: 2.18–3.35)	2.79 (2.478–3.143)	< 0.01	2.777 (2.462–3.135)	< 0.01	2.755 (2.442–3.11)	< 0.01	2.709 (2.398–3.059)	< 0.01
	Quartile 4 (VAI: 3.35–35.4)	5.374 (4.769–6.063)	< 0.01	5.341 (4.726–6.042)	< 0.01	5.261 (4.653–5.954)	< 0.01	5.138 (4.540–5.816)	< 0.01
Elevated TG	VAI (continuous)	1.536 (1.494–1.580)	< 0.01	1.594 (1.549–1.642)	< 0.01	1.588 (1.543–1.636)	< 0.01	1.588 (1.542–1.635)	< 0.01
	Quartile 1 (VAI: 0.242–1.40)	Ref	Ref	Ref	Ref	Ref	Ref	Ref	Ref
	Quartile 2 (VAI: 1.40–2.18)	1.491 (1.314–1.694)	< 0.01	1.659 (1.458–1.889)	< 0.01	1.651 (1.451–1.879)	< 0.01	1.650 (1.450–1.879)	< 0.01
	Quartile 3 (VAI: 2.18–3.35)	4.008 (3.551–4.528)	< 0.01	4.851 (4.277–5.51)	< 0.01	4.814 (4.244–5.469)	< 0.01	4.827 (4.251–5.482)	< 0.01
	Quartile 4 (VAI: 3.35–35.4)	10.418 (9.174–11.847)	< 0.01	13.251 (11.583–15.184)	< 0.01	13.063 (11.415–14.973)	< 0.01	13.121 (11.451–15.035)	< 0.01
Elevated WC	VAI (continuous)	1.086 (1.065–1.108)	< 0.01	1.042 (1.023–1.063)	< 0.01	1.04 (1.02–1.06)	< 0.01	1.04 (1.002–1.06)	< 0.01
	Quartile 1 (VAI: 0.242–1.40)	Ref	Ref	Ref	Ref	Ref	Ref	Ref	Ref
	Quartile 2 (VAI: 1.40–2.18)	1.333 (1.195–1.486)	< 0.01	1.152 (1.029–1.289)	0.014	1.145 (1.024–1.282)	0.018	1.151 (1.028–1.288)	0.014
	Quartile 3 (VAI: 2.18–3.35)	1.608 (1.441–1.795)	< 0.01	1.271 (1.134–1.426)	< 0.01	1.26 (1.124–1.413)	< 0.01	1.270 (1.132–1.424)	< 0.01
	Quartile 4 (VAI: 3.35–35.4)	1.863 (1.668–2.083)	< 0.01	1.412 (1.257–1.586)	< 0.01	1.389 (1.236–1.561)	< 0.01	1.394 (1.240–1.567)	< 0.01
Elevated BP	VAI (continuous)	1.04 (1.027–1.06)	< 0.01	1.051 (1.032–1.07)	< 0.01	1.043 (1.024–1.062)	< 0.01	1.044 (1.025–1.063)	< 0.01
	Quartile 1 (VAI: 0.242–1.40)	Ref	Ref	Ref	Ref	Ref	Ref	Ref	Ref
	Quartile 2 (VAI: 1.40–2.18)	1.146 (1.023–1.284)	0.019	1.154 (1.028–1.297)	0.016	1.135 (1.01–1.275)	0.034	1.137 (1.011–1.278)	0.032
	Quartile 3 (VAI: 2.18–3.35)	1.285 (1.148–1.438)	< 0.01	1.318 (1.173–1.482)	< 0.01	1.282 (1.14–1.442)	< 0.01	1.290 (1.146–1.451)	< 0.01
	Quartile 4 (VAI: 3.35–35.4)	1.402 (1.253–1.568)	< 0.01	1.46 (1.299–1.642)	< 0.01	1.388 (1.233–1.562)	< 0.01	1.403 (1.246–1.580)	< 0.01

### 3.5 Subgroup Analyses of VAI–MetS Association Across Demographic and Lifestyle Factors

**FIGURE 2:**
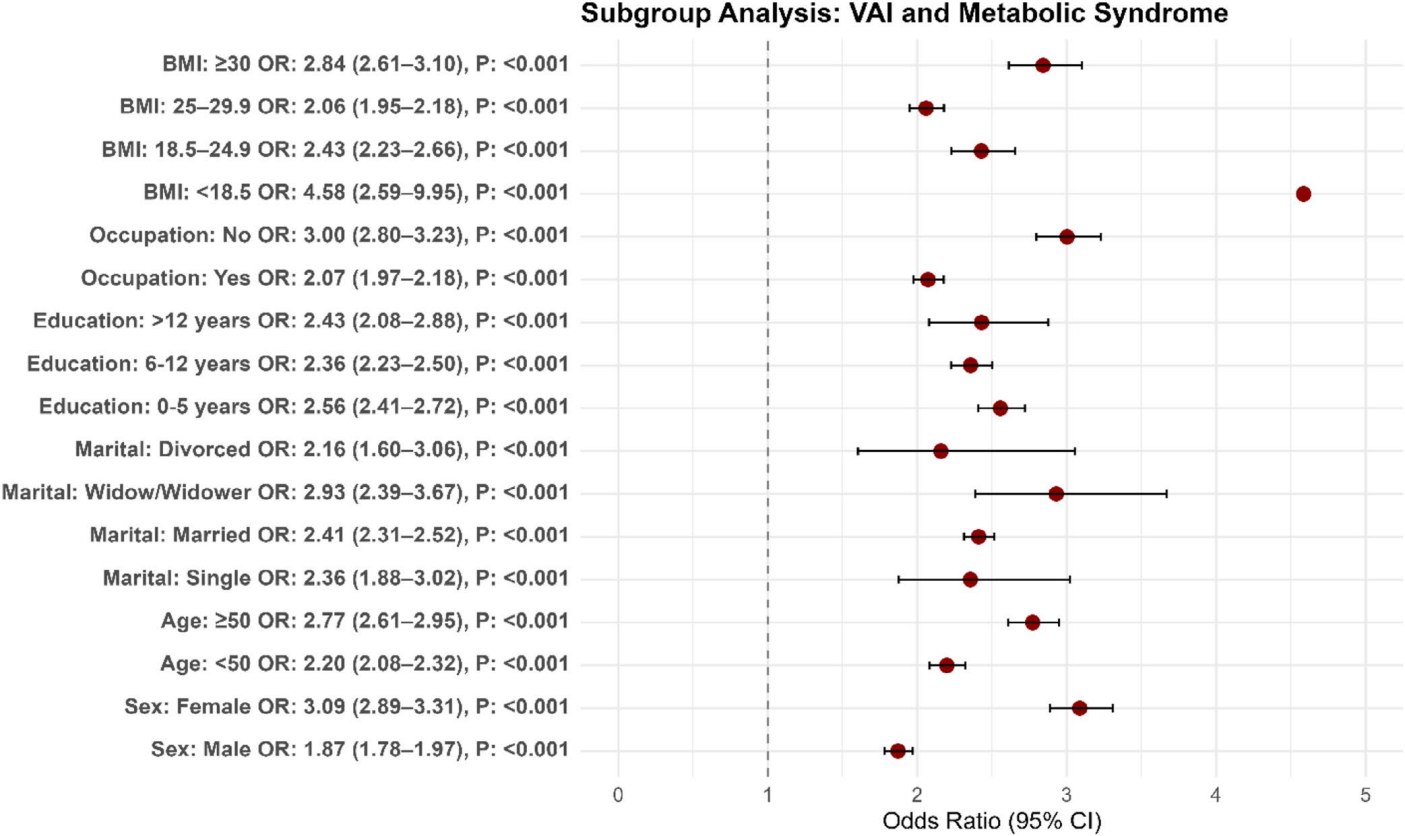
Subgroup analysis of the association between VAI and MetS across demographic and clinical characteristics based on crude model.

To examine the consistency of the association between the VAI and MetS, stratified analyses were performed across selected demographic and lifestyle subgroups. In all examined strata—including sex, age, marital status, education, occupation and BMI categories—a positive association between VAI and the odds of MetS was observed. The direction and magnitude of the association remained stable, indicating robustness of the findings across diverse population segments. (Figure 2).

## 4 Discussion

### 4.1 Clinical Relevance

In this large population‐based study from the PGCS dataset, higher VAI was consistently associated with greater odds of the presence of MetS in the overall population and in both men and women. This association remained statistically significant after adjustment for demographic, lifestyle and clinical covariates. Further analysis indicated that the magnitude of the association significantly differed by sex, with a stronger association observed among women.

VAI also showed good discriminatory ability for identifying MetS within the study population. Among individual MetS components, the strongest associations were observed with elevated TG and low HDL‐C, which are incorporated in the VAI formula. Nevertheless, VAI was also significantly associated with elevated FPG and elevated BP, suggesting that the association between VAI and MetS may not be limited only to its shared lipid and anthropometric components. Furthermore, subgroup analyses across age, education and BMI supported the consistency of the observed associations across examined population subgroups. Taken together, these results may suggest the potential value of VAI as a clinically practical and accessible tool for early cardiometabolic risk assessment.

### 4.2 Comparison With Previous Studies

Several studies have explored the role of VAI in relation to MetS. Amato et al., who first introduced the VAI, demonstrated that it was a reliable surrogate marker of visceral fat function and a stronger marker of cardiometabolic risk than traditional measures such as BMI or WC [11]. Their work underscored the role of visceral fat dysfunction—particularly lipid derangements—in the pathogenesis of MetS.

Expanding upon this foundational evidence, a meta‐analysis by Bijari et al. demonstrated that VAI has good discriminatory accuracy for detecting MetS, with a pooled AUC of 0.847, sensitivity of 78% and specificity of 79% [21]. These findings are consistent with our results, in which VAI showed good discriminatory ability for identifying MetS in the study population. In line with these findings, Vicente Herrero et al. examined Spanish workers and confirmed that both VAI and DAI (Dysfunctional Adiposity Index) had good discriminatory performance across multiple MetS definitions (ATP III, IDF, JIS), with VAI performing best under the JIS criteria [22]. These results further support the relevance of VAI as a cardiometabolic risk marker in large and diverse working populations.

Beyond MetS in general population settings, a growing body of research has examined VAI in the context of other metabolic and cardiometabolic conditions, especially among high‐risk groups [23, 24, 25]. For instance, Rocha et al. found that VAI was the index with the highest discriminatory performance for identifying MetS in women with polycystic ovary syndrome (PCOS), surpassing Lipid Accumulation Product (LAP) and HOMA‐IR. Among PCOS patients with MetS, 92.3% had abnormal VAI values, and ROC analysis supported its favourable discriminatory performance [26]. Similarly, Fahmy et al. assessed VAI and LAP in 209 Egyptian patients with chronic kidney disease. While LAP slightly outperformed VAI overall, VAI remained significantly associated with insulin resistance and MetS, especially in male patients (AUC = 0.839). This highlights its relevance even in comorbid populations [27].

In individuals with suspected obstructive sleep apnea (OSA), Chen et al. reported that VAI levels increased with OSA severity and were strongly linked to MetS. A VAI threshold ≥ 2.282 identified over a 10‐fold increased risk of MetS, with an AUC of approximately 0.83 [28]. Further supporting these findings, Lazzer et al. evaluated five adiposity indices in 876 women with severe obesity and found that VAI and CMI significantly outperformed WHR, WtHR and BMFI. VAI demonstrated good discriminatory power (AUC = 0.84) and showed strong correlations with HDL‐C and TG [29], which is in line with our results demonstrating that VAI had the strongest associations with the dyslipidemic components of MetS.

These findings collectively support the relevance of VAI across diverse high‐risk populations, including those at risk for cardiovascular and cerebrovascular events, diabetes, renal impairment, hormonal disorders, sleep‐disordered breathing and severe obesity. Notably, the consistent performance of VAI in identifying MetS across heterogeneous groups highlights its potential versatility as a cardiometabolic risk marker. Our study further contributes to this growing body of evidence by demonstrating good discriminatory ability of VAI in a large general population dataset.

Previous comparative studies have also suggested that VAI may perform favourably compared with several conventional adiposity indices, although findings vary by population, diagnostic criteria and comparator index. For example, Al‐Daghri et al. assessed six adiposity indices—including VAI—for their ability to detect cardiometabolic diseases in two large Saudi cohorts (*N* = 6821), using the International Diabetes Federation (IDF) criteria. VAI exhibited the highest sensitivity for predicting both type 2 diabetes and MetS in at least one cohort (AUC = 0.837), with better performance than BMI and WHR. Its strong correlations with triglycerides and HDL‐C further underscore its metabolic relevance, even among non‐Caucasian populations under ethnically adjusted MetS definitions [30].

Furthermore, Gu et al. compared five indices—BMI, WHtR, TG/HDL‐C, LAP and VAI—among 6722 elderly Chinese adults using IDF criteria. While LAP had the highest AUC overall, VAI still showed good diagnostic performance (AUC = 0.865 in men, 0.856 in women) and remained independently associated with the accumulation of MetS components, reinforcing its potential relevance across different demographic groups [31].

Although our study focused on middle‐aged adults (35–70 years), recent evidence has confirmed that VAI remains a marker associated with MetS in younger age groups as well. For instance, Vega‐Cárdenas et al. [32] studied 1372 young adults and found that VAI had the highest discriminatory accuracy among several indices (AUC = 0.932), supporting its potential relevance even in late adolescence. Similarly, Vizzuso et al. [33] evaluated 637 obese children and adolescents (8–15 years) and confirmed VAI as the best anthropometric marker associated with MetS compared to BMI *z*‐score, ABSI and WHR, with ROC‐AUCs around 0.77–0.80 depending on sex. These findings suggest that VAI has shown favourable performance compared with conventional indices in some populations for early cardiometabolic risk stratification, regardless of age group.

Taken together, previous studies suggest that VAI may perform better compared to several traditional indices across different age groups and populations. In the present study, exploratory ROC analyses showed that VAI had higher discriminatory ability for identifying MetS than BMI, WC, WHtR and the TG/HDL‐C ratio in the overall population. This finding suggests that the composite structure of VAI, which incorporates both anthropometric and lipid‐related parameters, may capture cardiometabolic risk more effectively than individual anthropometric measures or the lipid ratio alone. However, these comparisons were descriptive and conducted within the same study population; therefore, future studies with external validation are needed to clarify whether VAI provides added value beyond routinely available indices.

### 4.3 Clinical Implication

From a clinical and public health perspective, VAI is based on routinely available anthropometric and biochemical measurements and may serve as a simple marker of cardiometabolic risk in population‐based settings. In the present study, VAI showed good discriminatory ability for identifying MetS, with optimal cut‐off values of 2.42 in the overall population, 2.08 in men and 2.69 in women. However, these thresholds were derived and evaluated in the same dataset and should therefore be considered exploratory. Further prospective studies with external validation are required before these cut‐off values can be recommended for clinical prediction or screening.

### 4.4 Strengths and Limitations

Despite the notable strengths of our study—including the use of a large, population‐based sample drawn from the PERSIAN Guilan Cohort, detailed demographic and clinical data, and rigorous statistical adjustment for a wide range of potential confounders—several limitations should be acknowledged. The cross‐sectional design inherently limits the ability to infer causality between VAI and the development of MetS or its individual components. Therefore, the findings should be interpreted as associations with prevalent MetS rather than evidence of prospective prediction. Longitudinal studies are needed to determine the temporal relationship and predictive power of VAI for incident cardiometabolic outcomes.

Second, an important methodological consideration is the partial mathematical overlap between VAI and the ATP III definition of MetS. VAI incorporates WC, triglycerides and HDL‐C, which are also included as MetS criteria. This overlap may partly contribute to the magnitude of the observed associations and the discriminatory performance of VAI. For this reason, the large odds ratios, particularly those observed in the highest VAI quartile, should be interpreted cautiously and should not be viewed as reflecting a purely independent biological effect. Nevertheless, VAI was also associated with elevated FPG and elevated BP, which are not included in the VAI equation, suggesting that its association with MetS may extend beyond the shared lipid and anthropometric components.

Third, VAI had a right‐skewed distribution with a broad upper range. This skewness may affect the interpretation of continuous estimates and quartile‐based comparisons. To reduce reliance on distributional assumptions, we evaluated the association between VAI and MetS using both continuous and categorical quartile‐based analyses. While the overall association observed in continuous models was not driven exclusively by the highest VAI category, estimates related to the upper quartile should nonetheless be interpreted with caution.

Fourth, although anthropometric measurements were collected using standardized protocols, a small number of participants exhibited extreme values of BMI, reflecting outliers in weight and height. These observations were retained in the analysis to preserve the population‐based nature of the cohort and avoid selection bias. In addition, although exploratory ROC analyses compared VAI with selected routinely available indices, including BMI, WC, WHtR and the TG/HDL‐C ratio, these comparisons were descriptive and conducted within the same study population. Therefore, the ROC‐derived cut‐off values and comparative discriminatory findings should be considered exploratory and require external validation.

Finally, the study's methodological robustness, sex‐stratified and subgroup analyses, and consistent findings across multiple models add strength and credibility to the observed associations. The findings are particularly relevant for low‐ and middle‐income countries, where cost‐effective markers like VAI may have value in early cardiometabolic risk assessment. Future prospective studies are warranted to validate these results in other populations and to clarify the longitudinal value of VAI for cardiovascular and metabolic outcomes.

## 5 Conclusion

This study demonstrated that VAI was strongly associated with the presence of MetS in a large Iranian adult population and showed good discriminatory ability within the study population. Among components, the strongest associations were observed with elevated TG and low HDL‐C. Although these findings support the relevance of VAI as an accessible marker of cardiometabolic risk, the cross‐sectional design and derivation of cut‐off values within the same dataset should be considered when interpreting the results. Further prospective studies with external validation are needed to determine the value of VAI for risk assessment and clinical application.

## Author Contributions

**Negin Letafatkar:** conceptualization, data curation, formal analysis, visualization, writing – review and editing. **Ehsan Amini‐Salehi:** conceptualization, data curation, writing – original draft, writing – review and editing. **Seyyed Mohammad Hashemi:** writing – original draft, writing – review and editing, visualization. **Soheil Hassanipour:** formal analysis, investigation, data curation. **Maryam Jafari:** writing – original draft. **Saeed Ghaffari:** writing – original draft. **Farahnaz Joukar:** investigation, conceptualization, writing – review and editing. **Fariborz Mansour‐Ghanaei:** investigation, conceptualization, project administration.

## Funding

The authors have nothing to report.

## Ethics Statement

This study was conducted in accordance with the ethical standards of the Declaration of Helsinki. The study protocol was approved by the Ethics Committee of Guilan University of Medical Sciences (Ethics Code: IR.GUMS.REC.1405.014).

## Consent

Written informed consent was obtained from all participants prior to data collection.

## Conflicts of Interest

The authors declare no conflicts of interest.

## Data Availability

The datasets used and/or analysed during the current study are available from the corresponding author on reasonable request.
